# Supplementary material for: Self-organization, quality control, and preclinical studies of human iPSC-derived retinal sheets for tissue-transplantation therapy
Source: Commun Biol. 2023 Feb 10;6:164. doi: 10.1038/s42003-023-04543-5 (PMC9918541; doi:10.1038/s42003-023-04543-5)
Supplement: Supplementary file 2 — Description of Additional Supplementary Files [file 42003_2023_4543_MOESM2_ESM.pdf]

Description of Additional Supplementary Files

File name: Supplementary Data

Description: The source data for graphs in the paper.
