# Supplementary material for: Self-organization, quality control, and preclinical studies of human iPSC-derived retinal sheets for tissue-transplantation therapy
Source: Commun Biol. 2023 Feb 10;6:164. doi: 10.1038/s42003-023-04543-5 (PMC9918541; doi:10.1038/s42003-023-04543-5)
Supplement: Supplementary file 4 — Reporting Summary [file 42003_2023_4543_MOESM4_ESM.pdf]

nature portfolio

Corresponding author(s): Atsushi Kuwahara

Last updated by author(s): Jan 29, 2023

Reporting Summary

Nature Portfolio wishes to improve the reproducibility of the work that we publish. This form provides structure for consistency and transparency in reporting. For further information on Nature Portfolio policies, see our Editorial Policies and the Editorial Policy Checklist.

Statistics

For all statistical analyses, confirm that the following items are present in the figure legend, table legend, main text, or Methods section.

n/a	Confirmed	
☐	☒	The exact sample size (*n*) for each experimental group/condition, given as a discrete number and unit of measurement
☐	☒	A statement on whether measurements were taken from distinct samples or whether the same sample was measured repeatedly
☐	☒	The statistical test(s) used AND whether they are one- or two-sided<br>*Only common tests should be described solely by name; describe more complex techniques in the Methods section.*
☒	☐	A description of all covariates tested
☒	☐	A description of any assumptions or corrections, such as tests of normality and adjustment for multiple comparisons
☐	☒	A full description of the statistical parameters including central tendency (e.g. means) or other basic estimates (e.g. regression coefficient) AND variation (e.g. standard deviation) or associated estimates of uncertainty (e.g. confidence intervals)
☐	☒	For null hypothesis testing, the test statistic (e.g. *F*, *t*, *r*) with confidence intervals, effect sizes, degrees of freedom and *P* value noted<br>*Give P values as exact values whenever suitable.*
☐	☒	For Bayesian analysis, information on the choice of priors and Markov chain Monte Carlo settings
☒	☐	For hierarchical and complex designs, identification of the appropriate level for tests and full reporting of outcomes
☒	☐	Estimates of effect sizes (e.g. Cohen's *d*, Pearson's *r*), indicating how they were calculated

Our web collection on statistics for biologists contains articles on many of the points above.

Software and code

Policy information about availability of computer code

Data collection	Image analyses were performed with IMARIS (Oxford Instruments), ImageJ (National Institutes of Health), and Zen Blue (Carl Zeiss) imaging software.
Data analysis	Statistical analyses were performed with R version 3.6.0 (The R Foundation for Statistical Computing).

For manuscripts utilizing custom algorithms or software that are central to the research but not yet described in published literature, software must be made available to editors and reviewers. We strongly encourage code deposition in a community repository (e.g. GitHub). See the Nature Portfolio guidelines for submitting code & software for further information.

Data

Policy information about availability of data

All manuscripts must include a data availability statement. This statement should provide the following information, where applicable:

- Accession codes, unique identifiers, or web links for publicly available datasets
- A description of any restrictions on data availability
- For clinical datasets or third party data, please ensure that the statement adheres to our policy

All reasonable requests will be promptly reviewed by the senior authors to determine whether the request is subject to any intellectual property or confidentiality obligations. This study did not generate new cell lines. Source data for graphs is provided as Supplementary Data. Microarray transcriptome data are available with accession number GSE197446.

Human research participants

Policy information about studies involving human research participants and Sex and Gender in Research.

Reporting on sex and gender	*Use the terms sex (biological attribute) and gender (shaped by social and cultural circumstances) carefully in order to avoid confusing both terms. Indicate if findings apply to only one sex or gender; describe whether sex and gender were considered in study design whether sex and/or gender was determined based on self-reporting or assigned and methods used. Provide in the source data disaggregated sex and gender data where this information has been collected, and consent has been obtained for sharing of individual-level data; provide overall numbers in this Reporting Summary. Please state if this information has not been collected. Report sex- and gender-based analyses where performed, justify reasons for lack of sex- and gender-based analysis.*
Population characteristics	*Describe the covariate-relevant population characteristics of the human research participants (e.g. age, genotypic information, past and current diagnosis and treatment categories). If you filled out the behavioural & social sciences study design questions and have nothing to add here, write "See above."*
Recruitment	*Describe how participants were recruited. Outline any potential self-selection bias or other biases that may be present and how these are likely to impact results.*
Ethics oversight	*Identify the organization(s) that approved the study protocol.*

Note that full information on the approval of the study protocol must also be provided in the manuscript.

Field-specific reporting

Please select the one below that is the best fit for your research. If you are not sure, read the appropriate sections before making your selection.

☒ Life sciences ☐ Behavioural & social sciences ☐ Ecological, evolutionary & environmental sciences

For a reference copy of the document with all sections, see nature.com/documents/nr-reporting-summary-flat.pdf

Life sciences study design

All studies must disclose on these points even when the disclosure is negative.

Sample size	No statistical methods were used to predetermine sample size.
Data exclusions	No data were excluded from analysis.
Replication	Each experiment was performed with at least three biological replicates.
Randomization	All samples were randomly allocated into experimental groups.
Blinding	No formal blinding was used.

Reporting for specific materials, systems and methods

We require information from authors about some types of materials, experimental systems and methods used in many studies. Here, indicate whether each material, system or method listed is relevant to your study. If you are not sure if a list item applies to your research, read the appropriate section before selecting a response.

Materials & experimental systems

n/a	Involved in the study	
☐	☒	Antibodies
☐	☒	Eukaryotic cell lines
☒	☐	Palaeontology and archaeology
☐	☒	Animals and other organisms
☒	☐	Clinical data
☒	☐	Dual use research of concern

Methods

n/a	Involved in the study	
☒	☐	ChIP-seq
☒	☐	Flow cytometry
☒	☐	MRI-based neuroimaging

Antibodies

Antibodies used	anti-Chx10 (sheep; Exalpha), anti-Pax6 (mouse; BD Biosciences), anti-Rx (guinea pig; Takara), anti-Crx (rabbit; Takara), anti-Recoverin (rabbit; Proteintech), anti-RXRG (mouse; Santa Cruz Biotechnology), anti-NRL (goat; R&D Systems), anti-Lhx2 (rabbit; Millipore), anti-Rhodopsin (mouse; Sigma Aldrich), anti-S-opsin (goat; Santa Cruz Biotechnology), anti-S-opsin (rabbit; Santa Cruz Biotechnology), anti-L/M-opsin (rabbit; Millipore), anti-Cone-arrestin (Arrestin-3; goat; Novus), anti-S-arrestin (mouse; Novus), anti-CtBP2 (mouse; BD Biosciences), anti-PKCalpha (goat; R&D Systems), anti-Ki67 (mouse; BD Biosciences), anti-Aqp1 (Aquaporin1; rabbit; Millipore), anti-Emx2 (sheep; R&D Systems), anti-cleaved caspase-3 (rabbit; Cell Signaling Technology), anti-HuNu (mouse; Millipore), anti-human Ku80 (rabbit; Cell Signaling Technology), anti-human Ku80 (goat; R&D Systems), anti-Gnat1 (rabbit; Santa Cruz Biotechnology), anti-Gnat2 (rabbit; Santa Cruz Biotechnology), anti-PRPH2 (mouse; Millipore), anti-PNA-Alexa Fluor 647 (Thermo), anti-Synaptophysin (goat; R&D Systems), and anti-LRIT3 (rabbit; Novus).
Validation	Validation was performed by manufacturers.

Eukaryotic cell lines

Policy information about cell lines and Sex and Gender in Research

Cell line source(s)	Two human iPSC lines, iPSC-LPF11 and iPSC-S17, were established from peripheral blood cells using Sendai virus vectors by Sumitomo Pharma. The iPSC-QHJI01s04 line (iPSC-Q) was established by the iPSC Stock Project organized by the Kyoto University Center for iPS Cell Research and Application (CiRA), and provided from Kyoto University. The iPSC-1231A3 line established at Kyoto University and derived from ePBMC(R) purchased from Cellular Technology Limited was provided by Kyoto University.
Authentication	iPSC-QHJI01s04 line was authenticated by Kyoto University (Doi et al. Nat Commun 2020; Yoshida et al. Med 2022). iPSC-S17 was authenticated by the short tandem repeat (STR) pattern analysis. Other cell lines, iPSC-LPF11 and iPSC-1231A3, were not authenticated.
Mycoplasma contamination	All cell lines are negative for mycoplasma contamination.
Commonly misidentified lines (See ICLAC register)	N/A

Animals and other research organisms

Policy information about studies involving animals; ARRIVE guidelines recommended for reporting animal research, and Sex and Gender in Research

Laboratory animals	RD-nude rats (SD-Foxn1 Tg(S334ter)3Lav nude rats; obtained from the Rat Resource and Research Center) and nude rats (F344/NJcl-rnu/rnu rats; CLEA Japan) were used for the study.
Wild animals	The study did not involve wild animals.
Reporting on sex	In the tumorigenicity study, female nude rats were used to reduce fighting between rats bred in a same cage.
Field-collected samples	The study did not involve samples collected from the field.
Ethics oversight	In vivo tumorigenicity study using animals was approved by the IRB of the Foundation for Biomedical Research and Innovation (FBRI), the Committee for Animal Experiments of the FBRI, and the animal care committee of RIKEN Center for Biosystems Dynamics Research (BDR). All the animal experimental protocols were approved by the animal care committee of the RIKEN BDR and were conducted in accordance with the Association for Research in Vision and Ophthalmology Statement for the Use of Animals in Ophthalmic and Vision Research.

Note that full information on the approval of the study protocol must also be provided in the manuscript.
